# Common germline-somatic variant interactions in advanced urothelial cancer

**DOI:** 10.1038/s41467-020-19971-8

**Published:** 2020-12-03

**Authors:** Aram Vosoughi, Tuo Zhang, Kyrillus S. Shohdy, Panagiotis J. Vlachostergios, David C. Wilkes, Bhavneet Bhinder, Scott T. Tagawa, David M. Nanus, Ana M. Molina, Himisha Beltran, Cora N. Sternberg, Samaneh Motanagh, Brian D. Robinson, Jenny Xiang, Xiao Fan, Wendy K. Chung, Mark A. Rubin, Olivier Elemento, Andrea Sboner, Juan Miguel Mosquera, Bishoy M. Faltas

**Affiliations:** 1 Department of Pathology and Laboratory Medicine, Weill Cornell Medicine, New York, NY USA; 2 Caryl and Israel Englander Institute for Precision Medicine, Weill Cornell Medicine-New York-Presbyterian Hospital, New York, NY USA; 3 Genomic Resources Core Facility, Weill Cornell Medicine, New York, NY USA; 4 Department of Medicine, Division of Hematology and Medical Oncology, Weill Cornell Medicine, New York, NY USA; 5 Department of Clinical Oncology, Kasr Alainy School of Medicine, Cairo University, Cairo, Egypt; 6 Institute for Computational Biomedicine, Weill Cornell Medicine, New York, New York, NY USA; 7 Division of Medical Oncology, Dana Farber Cancer Institute, Boston, MA USA; 8 Department of Pathology, Dartmouth–Hitchcock Medical Center, Lebanon, NH USA; 9 Departments of Pediatrics and Medicine, Columbia University, NY, Columbia, NY USA; 10 Department for Biomedical Research, University of Bern, Bern, Switzerland; 11 Department of Cell and Developmental Biology, Weill Cornell Medicine, New York, NY USA

**Keywords:** Cancer genomics, Bladder cancer

## Abstract

The prevalence and biological consequences of deleterious germline variants in urothelial cancer (UC) are not fully characterized. We performed whole-exome sequencing (WES) of germline DNA and 157 primary and metastatic tumors from 80 UC patients. We developed a computational framework for identifying putative deleterious germline variants (pDGVs) from WES data. Here, we show that UC patients harbor a high prevalence of pDGVs that truncate tumor suppressor proteins. Deepening somatic loss of heterozygosity in serial tumor samples is observed, suggesting a critical role for these pDGVs in tumor progression. Significant intra-patient heterogeneity in germline-somatic variant interactions results in divergent biological pathway alterations between primary and metastatic tumors. Our results characterize the spectrum of germline variants in UC and highlight their roles in shaping the natural history of the disease. These findings could have broad clinical implications for cancer patients.

## Abstract (web summary)

The role of germline variation in human cancers is not fully understood. Here, the authors define the landscape of putative deleterious germline variants that abrogate tumor suppressor proteins in advanced urothelial cancer patients.

## Introduction

Germline variants transmit genetic information that determines the heritability of complex disorders^1^. A previous study of urothelial cancer (UC) in twins showed significant heritability of up to 33%^2^. Recent work using targeted sequencing of known cancer susceptibility genes revealed a 14–24%^3,4^ prevalence of germline variants in UC patients, which accounts for only a fraction of the genetic predisposition for the disease. Individually-rare but collectively common germline variants can explain a substantial fraction of the missing genetic predisposition to UC^1^.

**Fig. 1:**
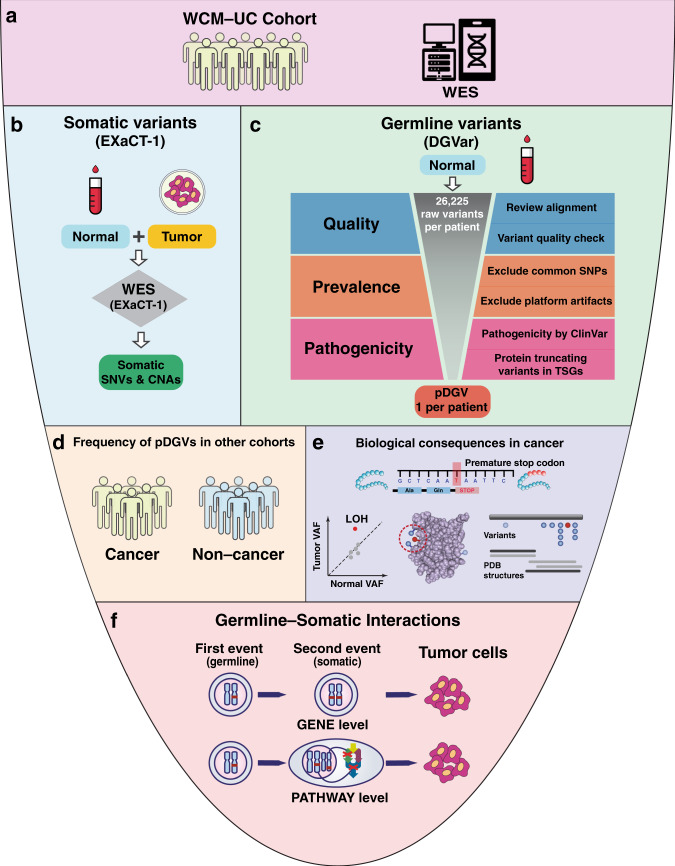
Comprehensive analysis of germline-somatic interactions in urothelial cancer. **a** WES of 157 germline samples from 80 UC patients (WCM-UC cohort) **b** Somatic variants identified through EXaCT-1 whole-exome sequencing (WES) pipeline using matched tumor-normal samples. **c** The DGVar framework for the identification of putative deleterious germline variants (pDGVs). **d** Comparison cohorts: 398 patients from the TCGA-BLCA cohort, and 11,035 noncancer subjects from the SPARK non-cancer cohort. **e** Functional predictions using CADD scores, three-dimensional modeling of the effects of pDGVs on protein data bank (PDB) structures and somatic LOH analysis **f** Germline-somatic interactions at the gene and pathway levels.

**Fig. 2:**
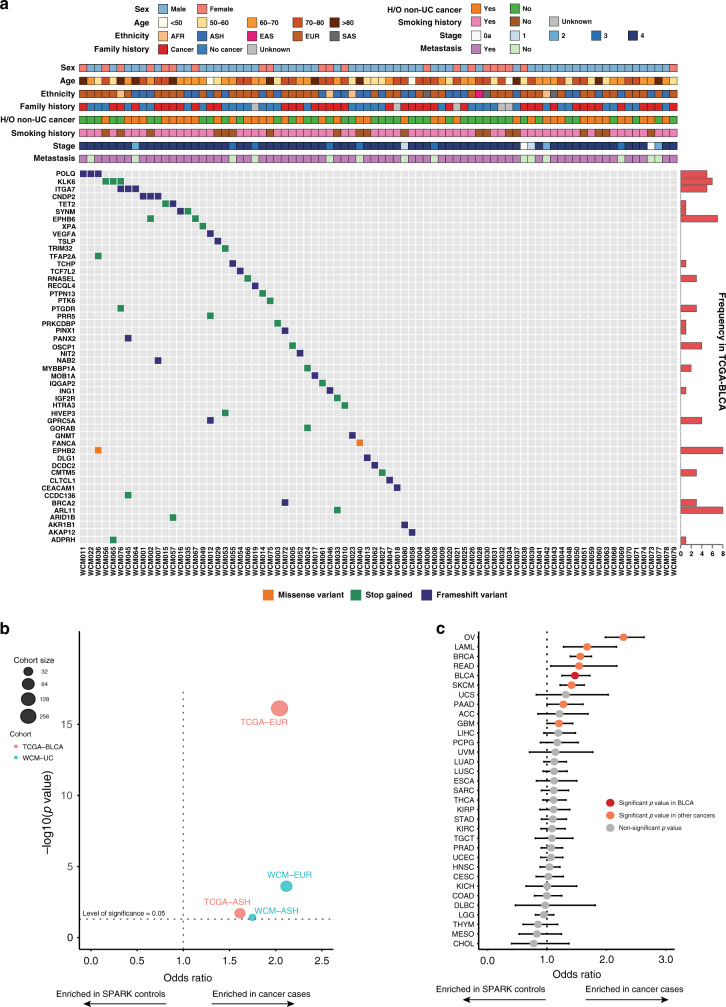
Putative deleterious germline variants are common in urothelial cancer patients. **a** pDGVs in the WCM-UC cohort. The frequencies of pDGVs in the same gene in the TCGA-BLCA cohort are displayed as horizontal bar plots (right). **b** The odds ratio of pDGVs to rare synonymous variants in a gene set of 158 genes comparing WCM-UC and TCGA-BLCA cancer cohorts to ethnicity-matched SPARK non-cancer cohorts using a two-sided Fisher’s exact test. Each circle corresponds to one of four comparisons: WCM-EUR vs. SPARK-EUR, WCM-AJ vs. SPARK-AJ, TCGA-EUR vs. SPARK-EUR, or TCGA-AJ vs. SPARK-AJ. Each circle’s diameter indicates the number of individuals in either the WCM-UC (blue) or TCGA-BLCA (red) cohorts. The horizontal dotted line indicates the statistical significance threshold above which the p-values are less than 0.05. The vertical dotted line represents an odds ratio of 1. Data points on the right have a higher ratio of pDGVs to rare synonymous variants in the WCM-UC and TCGA-BLCA cohorts. **c** The odds ratio of pDGVs to rare synonymous variants in a gene set of 158 genes comparing TCGA pan-cancer cohorts (*n* = 7,839) to SPARK non-cancer cohort (*n* = 11,035) with a two-sided Fisher’s exact test. Each circle indicates the odds ratio (OR), and the error bars indicate the 95% confidence intervals (CI). The vertical dotted line represents an odds ratio of 1. Values to the right of this line represent a higher odds ratio of pDGVs to rare synonymous variants in respective cancer cohorts compared to the SPARK non-cancer cohort. Source data are provided as a Source Data file.

To define the spectrum of germline variants affecting protein-coding genes and germline-somatic interactions (GSIs) in UC patients, we performed WES of prospectively collected germline DNA samples and 157 tumors from 80 UC patients at Weill Cornell Medicine (WCM-UC cohort) (Figs. 1a, 2a, and Supplementary Data 1). The majority of patients (82.5%) had metastatic disease during the study period. We developed a stepwise computational framework (DGVar) to distinguish putative deleterious germline variants (pDGVs) from a large number of background germline variants in each UC patient (Fig. 1b, c). To increase the specificity of this approach, we restricted our computational predictions to highly damaging events. To focus on functionally consequential germline variants, we adopted an approach to identify and prioritize germline variants that truncate tumor suppressor proteins. We then used DGVar to analyze germline WES data from 398 TCGA bladder cancer (TCGA-BLCA) cohort. We compared the pDGVs in the WCM-UC and TCGA-BLCA cohorts to an independent cohort of 11,035 ethnicity-matched noncancer subjects (Fig. 1d). We investigated the biological impact of pDGVs in UC tumors by screening three-dimensional protein structures for mutational clusters harboring pDGVs and somatic variants within the same domain (Fig. 1e). We examined loss of heterozygosity (LOH) events to identify pDGVs undergoing positive selection in the context of the two-hit model^5–8^ (Fig. 1e). To dissect the effects of pDGVs on UC throughout its lifetime, we examined LOH events in matched primary and metastatic tumors within the same patient. Finally, we interrogated specific GSIs occurring at the gene and pathway levels (Fig. 1f) to identify private alterations in distinct biological processes in individual UC tumors. Our results provide an atlas of pDGVs and define the spectrum of GSIs in UC patients.

## Results

### Development of a computational framework for identifying putative deleterious germline variants

We reasoned that variants that truncate tumor suppressor proteins would increase predisposition to cancer and potentially play an important role in tumor progression in the context of the classical two-hit model^5–8^. To identify these variants, we developed a computational framework (DGVar) that applies stringent criteria to germline sequencing data, including several quality checks to remove sequencing artifacts and exclude common single nucleotide polymorphisms (SNPs) reported in population databases (Online Methods). DGVar filtered out variants with inadequate read coverage (<10x), single-nucleotide variants (SNVs) with potential alignment problems, and variants that are commonly observed in the general population (>1% in ExAC). Most importantly, we restricted our definition of pDGVs to variants designated as pathogenic or likely pathogenic by ClinVar or those truncating proteins encoded by known tumor suppressor genes (TSGs) annotated in the COSMIC^9^ or TSGene^10^ lists (Online Methods) (Fig. 1). We included protein-truncating variants (stop gain or frameshift) that pass the inbreeding coefficient and variant quality score recalibration (VQSR) filters in ExAC (Online Methods). DGVar filtered a median of 26,225 raw germline variants per patient in the WCM-UC cohort to identify a median of one pDGV per patient (Fig. 1 and Supplementary Fig. 1a, b).

### Deleterious germline variants are common in urothelial cancer patients

We performed WES of germline DNA from 80 UC patients in our WCM cohort. WES data were analyzed using DGVar (Figs. 1a, 2a, and Supplementary Data 1). Most patients (59/80 (74%)) were male and (66/80 (82.5%)) had metastatic disease. The majority of patients (61/80 (76%)) had a history of smoking, 39 patients (49%) had a history of a second non-UC primary cancer, and 40 patients (50%) had a family history of cancer in at least one first-degree relative (Supplementary Data 1). The familial history of cancer rates reported in our cohort were consistent with previous reports^11,12^. Computational genomic ethnicity analysis using EthSEQ^13^ (Online Methods) showed a high representation of European (72/80 (90%)) and Ashkenazi Jewish (27/80 (34%)) ancestry in our cohort (Fig. 2a and Supplementary Data 1). We identified sixty-one germline pDGVs in 45 (56%) of patients in the WCM-UC cohort (Supplementary Data 2) (Online Methods). As expected, all pDGVs occurred in genes annotated as TSGs in the COSMIC^9^ or TSGene^10^ lists (Supplementary Data 3) (Online Methods). Out of 61 pDGVs identified in the WCM-UC cohort, 57 were not included in the cancer susceptibility genes (CSGs) list curated by Huang et al.^8^ or tested by a commercial targeted sequencing panel of 47 genes associated with cancer syndromes^14^ (Supplementary Fig. 2a, and Supplementary Data 2 and 3).

To validate our findings in a separate UC cohort, we used DGVar to analyze the germline WES data from the TCGA bladder cancer study (TCGA-BLCA). We identified 315 pDGVs in 48% (190/398) of patients in this cohort (Supplementary Data 4). In the WCM-UC cohort, *ITGA7, POLQ, KLK6, EPHB6*, and *CNDP2* were the most frequent genes harboring recurrent pDGVs, occurring in 11/45 patients (24%) (Fig. 2a and Supplementary Data 5). In the TCGA-BLCA cohort, 46 genes harbored recurrent pDGVs in 115/190 patients (60%) (Supplementary Data 5). We identified 12 pDGVs occurring in at least one patient in both the WCM-UC and TCGA-BLCA cohorts (Fig. 2a and Supplementary Data 5). The *EPHB6, ARL11, KLK6, ITGA7*, and *POLQ* genes harbored the most recurrent pDGVs in both cohorts (Fig. 2a and Supplementary Data 5). Pathway analysis showed an enrichment of pDGVs involving genes in the DNA repair pathway, including *POLQ, POLK, FANCA, XPA, ASCC1*, and *BRCA2* in 6/80 (7.5%) of WCM-UC patients (Supplementary Fig. 3). Twelve genes harboring pDGVs in the WCM-UC cohort were listed as causally implicated in cancer in the COSMIC Cancer Gene Census^15^ (https://cancer.sanger.ac.uk/census), and six genes (*BRCA2, FANCA, XPA, POLQ, PTPN13*, and *RECQL4*) were previously reported to harbor germline mutations in several cancer types (Supplementary Data 6). Out of 315 pDGVs identified by DGVar in the TCGA-BLCA cohort, 271 (85%) were not included in the CSGs or commercial testing gene lists (Supplementary Fig. 2b and Supplementary Data 4).

We hypothesized that pDGVs are enriched in UC patients compared to non-cancer subjects. We used the SPARK study^16^, which included whole-exome sequencing data from 11,035 adult non-cancer subjects of European (EUR) and Ashkenazi Jewish (AJ) ancestry for comparison. We calculated the ratio of pDGVs to rare synonymous variants in a gene set of 158 genes comparing ethnicity-matched urothelial cancer (WCM-UC and TCGA-BLCA) and non-cancer (SPARK) cohorts (Online Methods, Supplementary Data 3). The WCM-UC-EUR (Odds ratio (OR) = 2.12, *p* = 2.4e–4) and TCGA-BLCA-EUR (OR = 2.04, *p* = 7.4e–17) cancer patients were more likely to harbor pDGVs in this gene set compared to SPARK-EUR non-cancer subjects. Similarly, WCM-UC-AJ (OR = 1.75, *p* = 0.038) and TCGA-BLCA-AJ (OR = 1.61, *p* = 0.019) cancer patients were more likely to harbor pDGVs in this gene set compared to the SPARK-AJ non-cancer subjects (Online Methods) (Fig. 2b, Supplementary Data 7 and 8). We performed similar analyses of the TCGA pan-cancer and SPARK non-cancer cohorts. These comparisons were limited to individuals with self-reported white ethnicity in the TCGA pan-cancer cohorts. The TCGA-BLCA cohort was among the top five cancers with a significantly higher likelihood of harboring pDGVs (OR = 1.47, *p* = 3.42e–6) (Fig. 2c and Supplementary Data 9). Similarly, in an internal cohort of patients with non-UC, including prostate, breast, colorectal, kidney cancers, and glioblastoma, WCM-UC was the only cohort with a significantly higher likelihood of harboring pDGVs (WCM-UC-EUR OR = 2.12, *p* = 2.42e–4, and WCM-UC-AJ OR = 1.75, *p* = 0.038) (Supplementary Fig. 4 and Supplementary Data 10).

### The impact of pDGVs on protein structure and function

**Fig. 3:**
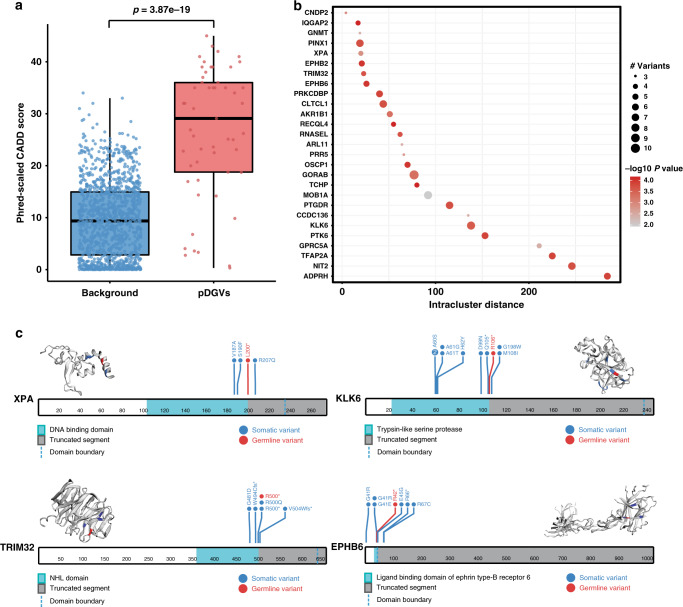
The impact of pDGVs on protein function. **a** Phred-scaled CADD scores are higher for pDGVs (*n* = 53 variants) than randomly selected control variants (*n* = 1249 variants), two-tailed Wilcoxon signed-rank test. The lower and upper edge of the box indicate the 25th and 75th percentiles. The black line in the center of the box indicates the median value; the lower and upper whiskers indicate 1.5 x the interquartile range **b** Bubble plot represents pDGV-somatic variant clusters for each gene. Circle diameters represent cluster sizes. The intra-cluster distances between amino acid positions are represented on the x-axis. -log10 *p*-values are represented as shades of red. **c** Lollipop plots showing the clustering of pDGVs, and somatic variants in *XPA, EPHB6, TRIM32*, and *KLK6* projected on their 3D protein structures. The truncated segment of each protein is shaded in gray. The boundary of the affected domain is delineated with a dashed line. WCM-UC pDGVs are colored in red, and known somatic variants are colored in blue. Source data are provided as a Source Data file.

To assess the potential deleteriousness of pDGVs, we compared the combined annotation dependent depletion (CADD)^17,18^ scores of pDGVs to background variants (Online Methods). CAAD makes a binary distinction between simulated de novo variants, which are possibly deleterious and neutral fixed variants that survive selective pressure^17,18^. As expected, pDGVs had significantly higher average CADD scores than randomly selected background variants (*p* = 3.9e–19) (Fig. 3a and Supplementary Data 11). Genomic variants that confer a fitness advantage on tumor cells tend to aggregate in functionally significant domains^19^. We used the Mutation3D^20^ tool to test whether pDGVs form distinct topological clusters with known somatic cancer mutations^21^ relative to the three-dimensional structures of the encoded proteins (Online Methods). Out of 28 pDGVs identified in the WCM-UC with available structural information for the encoded protein, 27 (96%) clustered with previously reported somatic variants (*p* < 0.001). These clusters harbored a median of 5 variants (Fig. 3b, c, and Supplementary Data 12) and frequently occurred in important domains (Fig. 3c). Six pDGVs in the *PINX1, MOB1A, CLTCL1, PRR5, CCDC136*, and *TRIM32* genes involved the exact amino acid residues affected by known somatic cancer variants (Supplementary Data 12).

**Fig. 4:**
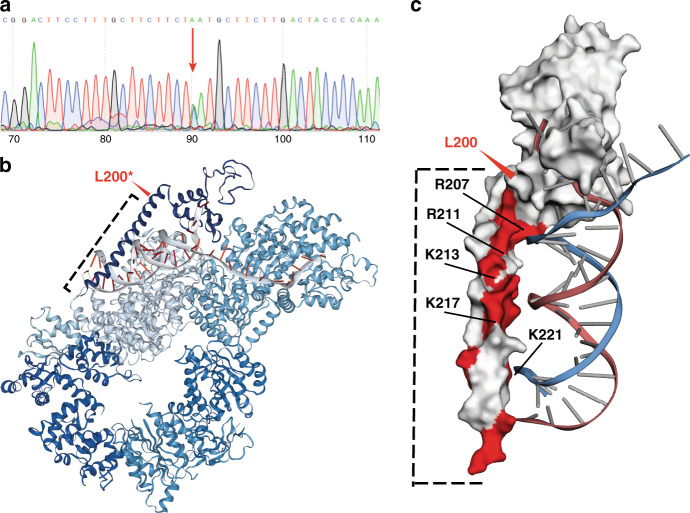
L200* eliminates the DNA-binding domain of XPA. **a** Sanger sequencing of germline DNA confirms the L200* pDGV in *XPA*. **b** The cryo-EM structure of the XPA-TFIIH complex showing the interaction between XPA and DNA during nucleotide excision repair^23^. The L200* pDGV (red arrow) eliminates the entire alpha-helix of the DNA binding domain (dotted line). **c** The L200* pDGV (red arrow) eliminates the positively charged amino acid residues (red) which bind to the negatively charged DNA backbone. These positively charged amino acid residues (arrows), such as R207, are commonly affected by germline mutations in xeroderma pigmentosum patients.

We identified a pDGV in the Xeroderma-Pigmentosum Group A-Complementing gene (*XPA*) gene in a UC patient. The patient did not have any clinical features of xeroderma pigmentosum apart from mild skin pigmentation and had not had previous germline testing. This pDGV resulted in an L200* stop codon clustered with other known somatic variants that target the DNA binding domain of XPA spanning codons 104–225 (Fig. 3c). It also clustered with previously identified pathogenic germline variants associated with clinical xeroderma pigmentosum, such as R207^22^ (Fig. 3c). We confirmed this variant’s presence using Sanger sequencing of the patient’s germline DNA (Fig. 4a). We also confirmed that this variant was expressed using RT-PCR of mRNA extracted from the patient’s tumor tissue (Supplementary Fig. 5) (Online Methods). The XPA protein is a part of a large multi-subunit complex, which has dual transcription factor and nucleotide-excision repair functions^23,24^. To predict the functional impact of the L200* XPA pDGV within this complex, we superimposed it on the recently published XPA-TFIIH complex structure obtained by cryogenic electron microscopy (cryo-EM)^24^ (Fig. 4b). This model predicts that L200* eliminates the entire DNA-binding alpha-helix domain of XPA. The deleted region contains 15 positively charged amino acids, including R207, R211, K213, K217, and K221, that interact with the negatively charged DNA backbone. This suggests that the L200* pDGV potentially causes significant disruption of DNA binding, which is required for nucleotide-excision repair^24,25^(Fig. 4c).

### Deepening loss of heterozygosity occurs under evolutionary pressure

**Fig. 5:**
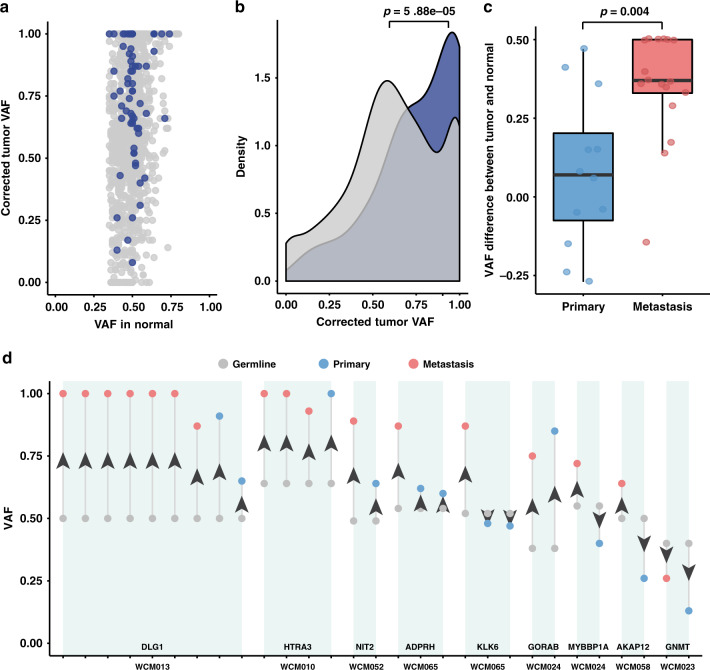
Deepening loss of heterozygosity during UC progression. **a** A scatter plot displaying the normal and tumor VAFs of pDGVs in TSGs (blue dots) and protein-truncating germline variants in non-TSGs (gray dots). The majority of pDGVs have a tumor-to-normal VAF ratio ≥1.6. **b** A density plot representing the distribution of the VAFs of pDGVs affecting TSGs and protein-truncating germline variants affecting non-TSGs. The peak density of the VAF of pDGVs in TSGs (blue) is significantly higher than the density of the VAF of protein-truncating germline variants in non-TSGs (gray) using a two-tailed Kolmogorov–Smirnov test. **c** The median VAF of pDGV is significantly higher in metastatic tumors (*n* = 17) compared to primary tumors (*n* = 12) in UC using a two-tailed Wilcoxon signed-rank test. The lower and upper edge of the box indicate the 25th and 75th percentiles. The black line in the center of the box indicates the median value; the lower and upper whiskers indicate 1.5x the interquartile range. **d** Changes in the VAFs of pDGVs in matched primary and metastatic UC trios. Metastatic, primary tumors, and germline are displayed in red, blue, and gray, respectively. Source data are provided as a Source Data file.

To gain insight into the functional role of pDGVs in UC progression, we hypothesized that loss-of-function pDGVs in TSGs undergo positive selection in UC tumors, which manifests as somatic loss of heterozygosity (LOH). LOH was defined as a tumor-to-normal variant allele frequency (VAF) ratio ≥ 1.6 (Online Methods). Indeed, we found that 53% of pDGVs showed evidence of LOH (Fig. 5a and Supplementary Data 13), and 34/72 (47%) of the tumor samples with sufficient purity had a corrected tumor-to-normal VAF ratio ≥1.6, indicating LOH (Supplementary Data 13) (Online Methods). The peak corrected tumor VAF density of pDGVs affecting TSGs was significantly higher compared to protein-truncating germline variants affecting non-TSGs (*p* = 5.9e–5), suggesting that LOH preferentially occurs in TSGs (Fig. 5b). As tumors are subject to continuous evolutionary pressures^26,27^, we posited that deepening LOH would occur as the cancer progresses from the primary to the metastatic state. We were uniquely positioned to study longitudinal pDGV LOH changes in the WCM-UC cohort, which included 29 primary and metastatic UC tumor pairs (Supplementary Data 14). We discovered that 79% (23/29) of the paired comparisons showed significant VAF increases in the metastatic tumors compared to the primary tumors (*p* = 0.004) (Fig. 5c, d) (Supplementary Data 14). These data suggest that the evolutionary pressure on pDGVs drives progressive LOH in metastatic UC and that pDGVs play a critical role in tumor progression consistent with the two-hit model^5–8^.

### Germline-somatic interactions in the biology of urothelial cancer

**Fig. 6:**
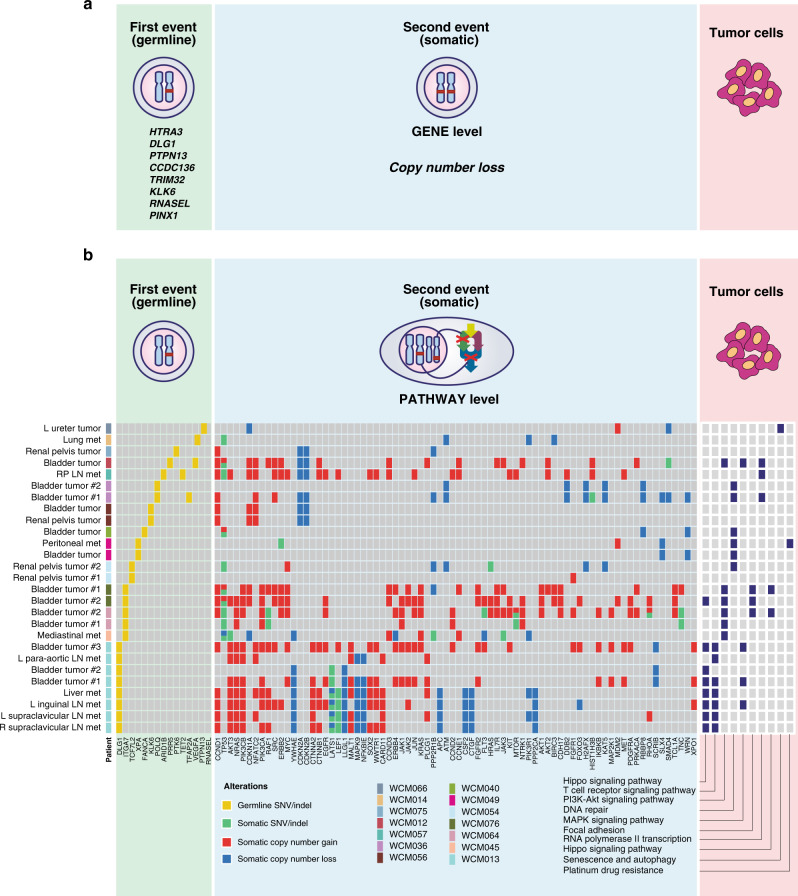
Germline and Somatic interactions in the urothelial cancer genome. **a** Schematic view of germline-somatic interactions at the gene level. Genes with somatic copy number losses were identified in eight WCM-UC patients. **b** Select pathway-level interactions between pDGVs and somatic genomic alterations in individual tumors from each patient. The complete list of somatic alterations is presented in Supplementary Fig. 8. Source data are provided as a Source Data file.

To define the mechanisms by which pDGVs contribute to UC progression, we examined GSIs occurring in the same gene (*in cis*) or other genes within the same biological pathway (*in trans*) (Fig. 6). We identified somatic copy number losses in 8/45 patients (18%) involving the *KLK6, HTRA3, DLG1, PTPN13, CCDC136, PINX1, RNASEL*, and *TRIM32* genes (Fig. 6a). We characterized pathway-level GSIs arising from the interaction of specific pDGVs with somatic mutations and copy-number variants of additional genes within a pre-defined biological pathway (Online Methods) (Fig. 6b and Supplementary Fig. 6). This analysis showed that 14 patients had at least one pathway-level GSI (*p* value < 0.05) (Supplementary Data 15), including GSIs in the DNA repair, *TP53* regulation, Hippo signaling, T-cell receptor signaling, and *WNT* signaling pathways (Fig. 6b) (Supplementary Data 15). We previously discovered extensive somatic intra-patient genomic heterogeneity arising from the clonal evolution of UC tumors^26^. We reasoned that this degree of somatic heterogeneity generates divergent GSIs in tumors within the same patient. In matched primary-advanced tumor pairs, we found that 60% of the tumors had GSIs in unique pathways that were not shared by other tumors from the same patient. These data collectively suggest that GSIs should be taken into consideration to understand the functional consequences of somatic alterations in cancer genomes.

## Discussion

Germline genomic integrity is safeguarded against high mutation rates^28^. When deleterious germline variants occur, they can have profound effects throughout an organism’s lifespan. For example, these variants can transmit genetic information that mediates hereditary cancer predisposition. Previous studies suggest that first-degree relatives of UC patients have a higher risk of developing UC^11^. A large epidemiological study of 203,691 individual twins estimated a 30% heritable component^2^. This was the same degree of heritability observed in breast cancer patients in the same study^2^. However, the germline determinants of increased UC risk are not fully characterized. Furthermore, the functional consequences of the majority of germline variants in UC biology are not well understood.

We sought to define the landscape of pDGVs that abrogate tumor suppressor proteins in advanced UC patients. We implemented a computational framework to identify pDGVs from WES data. Our findings suggest that pDGVs that are individually rare but collectively common, occurring in approximately half of UC patients. This is a significantly higher prevalence than previously thought^3,4,8,29^. The pDGVs identified in our study potentially explain a portion of the missing heritability of UC. Recent studies using targeted sequencing approaches showed that 7.3%–24% of UC patients carry pathogenic germline variants^3,4,8^. A recent study using targeted sequencing of 431 genes showed that the frequency of pathogenic germline variants in UC was 14%^4^. Another study using targeted sequencing of 42 genes identified 203 pathogenic germline variants in 24% of UC patients^3^. Our analysis suggests that targeted sequencing, which is frequently used for clinical testing approaches, significantly underestimates the prevalence of pDGVs in UC patients. This reflects the limited number of genes included in targeted panels. Our study demonstrates the feasibility of using whole-exome sequencing to interrogate a broader range of pDGVs in cancer patients.

Our computational framework has several distinctions from other approaches for germline variant detection^4,8^. We prioritized germline variants defined as pathogenic or likely pathogenic by ClinVar or those resulting in truncated proteins encoded by known TSGs. Our DGVar framework expands the definition of putative pathogenicity to include variants that eliminate critical domains of TSGs, likely resulting in loss of function.

We reasoned that germline variants that truncate tumor suppressor proteins would potentially predispose to UC and play a critical role in tumor progression in the context of the two-hit carcinogenesis model^5–8^. The majority of the pDGVs we identified clustered with known somatic variants within functional protein domains. Deepening LOH affecting the majority of pDGVs was observed during cancer progression, supporting their functional relevance. We identified a pDGV affecting exon 5 of *XPA* in a UC patient using WES and confirmed it using Sanger sequencing of the patient’s germline DNA. Functional modeling predicted that this pDGV (L200*) eliminates the protein’s DNA-binding domain critical for nucleotide-excision repair. The recently published cryo-EM structure positions XPA within the TFIIH complex at the edge of the DNA repair tunnel, suggesting that it plays a crucial role by attaching the core TFIIH complex to DNA^24^. An adjacent germline variant that affects the splice acceptor site in intron 3 and eliminates the c-terminus of XPA occurs in up to 1% of the Japanese population^30^. The exon 6 *XPA* germline variants R228* and H244R, which primarily affect the TFIIH-interacting region in the protein’s c-terminus, have been previously associated with a mild xeroderma pigmentosum phenotype^31,32^. Clinical and mouse model data suggest that heterozygous carriers of *XPA* mutations have a higher risk for developing cancer^23,30,33,34^. It is possible that the L200* truncating mutation we identified in *XPA* results in nonsense-mediated decay^35^ decreasing the relative abundance of the XPA protein.

We designed our approach to prioritize pDGVs in putative TSGs, including *KLK6*^36^, *EPHB6*^37,38^, and *TRIM32*^39^. We identified a *TRIM32* R500* pDGV that eliminated its NHL domain. Interestingly, a colocalizing somatic variant was found in a patient with endometrial carcinoma in the TCGA cohort^40^. TRIM32 is an E3 ubiquitin ligase that orchestrates the degradation of several targets^41^. Gli1, an effector of sonic hedgehog (SHH) signaling, binds to the NHL domain of TRIM32, resulting in degradation of the former^39^. Knockout of *Trim32* resulted in a higher incidence of medulloblastoma formation in the *Ptch1* ± mice and the upregulation of SHH target genes, suggesting a tumor suppressor effect from antagonizing SHH signaling^39^. Germline variants in other genes we identified, including *EPHB6* and *KLK6*, were reported in colorectal carcinoma^42^ and prostate cancer^43^. KLK6 re-expression in breast cancer cells reversed their malignant phenotype by inhibiting epithelial-to-mesenchymal transition^36^ consistent with a tumor suppressor role. EphB6 protein expression is differentially downregulated in invasive and metastatic breast cancer and causes a decrease in the invasiveness of breast cancer cell lines in vitro^38^. This is consistent with its role as a putative tumor suppressor. It is important to note that a given protein’s tumor suppressor function is lineage- and context-dependent^44,45^. Even canonical TSGs such as *TP53* can have oncogenic functions under specific circumstances^46,47^. High-throughput gene editing screens are beginning to generate direct experimental measurements of the pathogenicity of germline variants in different contexts^48^. Broader application of these approaches is expected to provide accurate pathogenicity data to inform clinical management.

Integrating germline and somatic genomic data can provide insights into the mechanisms that drive tumor progression^49^. We performed an in-depth integrated analysis of germline and somatic WES data in UC patients. First, we examined LOH, a hallmark of pDGV pathogenicity within the Knudson two-hit hypothesis, which suggests that most TSGs require inactivation of both alleles to cause a phenotypic change^5–8^. We observed a high rate of LOH affecting pDGVs in UC. A recent study showed that LOH patterns are tumor lineage-specific^50^. We observed progressive LOH in serial tumor samples in UC patients, suggesting that positive selection of pDGVs potentially plays role in UC progression. We identified significant intra-patient heterogeneity arising from private GSIs in individual tumors. These interactions involve divergent biological processes. Our findings highlight how germline-somatic variant interactions contribute to cancer heterogeneity. The functional consequences of these interactions warrant additional studies.

Our study was limited by sample size. To overcome this limitation, we analyzed 398 patients from the TCGA-BLCA cohort. We identified pDGVs in 48% of these patients confirming their high prevalence in UC patients. We used DNA extracted from peripheral blood mononuclear cells (PBMCs) for germline sequencing, it is possible that some of the pDGVs we detected resulted from clonal hematopoiesis of indeterminate potential (CHIP)^51,52^. However, none of the specific pDGVs we identified in our WCM-UC and TCGA-BLCA cohorts were previously identified as CHIP mutations^51,52^. The majority of pDGVs did not occur in genes commonly involved by CHIP^51,52^. The UC cohorts we studied had high representation of patients of European ancestry. We used ethnicity-matched non-cancer cohorts for comparison. The pDGVs profile is likely to be different in diverse populations. Germline studies can be particularly informative when somatic sequencing is insufficient to explain disparate clinical outcomes^53,54^.

Our findings have several important clinical implications. Consistent with previous studies^8,55–57^, we show that the WES expands the repertoire of germline variants beyond commonly used targeted sequencing approaches. While individually rare, pDGVs may be collectively common in cancer patients^56,57^. Our approach is generalizable to patients with other malignancies and likely to have a broad impact, given the growing use of WES in the clinic^58^. Recurrent pDGVs in DNA damage repair pathways are potential therapeutic targets. A randomized phase III study in patients with castrate-resistant prostate cancer recruited patients with alterations in the homologous recombination pathway^59^. Patients who received the PARP inhibitor olaparib had improved overall survival compared to those who received enzalutamide or abiraterone (HR 0.67, 95% CI 0.49–0.93). A recent study of Rucaparib in unselected UC patients showed stable disease in 28.4% of the patients^60^. Another study combining olaparib with immune checkpoint inhibition showed promising results in UC patients^61^. By expanding the repertoire of pDGVs in DNA damage repair genes, our results open the door to trials of these targeted therapeutic strategies in properly selected UC patients. In summary, our study characterized the spectrum of germline variants in UC. These findings have potential implications for precision medicine in thousands of UC patients.

## Methods

### Patient enrollment and tissue acquisition

All experimental procedures were carried out in accordance with approved guidelines and were approved by the Institutional Review Boards at WCM. Patients recruited to this study signed informed consent under IRB-approved protocols: WCM/New York-Presbyterian (NYP) IRB protocols for Tumor Biobanking—0201005295, GU tumor Biobanking—1008011210, Urothelial Cancer Sequencing—1011011386, Comprehensive Cancer Characterization by (Genomic and Transcriptomic Profiling—1007011157, and Precision Medicine—1305013903). Peripheral blood, buccal swab samples, and in one patient, normal liver tissue were collected for germline DNA extraction from 80 patients diagnosed with high-grade urothelial carcinoma (HGUC). Fresh frozen and formalin-fixed paraffin-embedded (FFPE) tissue from biopsies, cystectomy, and nephroureterectomy specimens from HGUC patients were collected^25^. All pathology specimens were reviewed and reported by board-certified genitourinary pathologists (AV, BDR, JMM, MAR) in the department of pathology at WCM/NYP. Clinical charts were reviewed by the authors (PJV, AV, BMF) to record patient demographics, tobacco use, family history of cancer, concurrent cancer, treatment history, anatomic site, pathologic grade, and stage using the tumor, node, metastasis (TNM) system.

### DNA extraction and whole-exome sequencing (WES)

For WCM-UC samples, our established Whole-Exome Sequencing (WES) protocol was used, as previously described^62,63^. Germline DNA was extracted using the Promega Maxwell 16 MDx (Promega, Madison, WI, USA), from peripheral blood mononuclear cell (PBMC) or buccal swab^25^, except for one patient whose sample was collected from a normal liver tissue obtained from an autopsy. Tumor DNA was extracted from a macrodissected target lesion from FFPE or cored OCT-cryopreserved tumors using the same method. Pathological review by one of the study pathologists (AV, BDR, JMM, MAR) confirmed the diagnosis and determined tumor content. A minimum of 200 ng of DNA was used for WES. The DNA quality was determined by TapeStation Instrument (Agilent Technologies, Santa Clara, CA) and was confirmed by real-time PCR before sequencing. Sequencing was performed with pair-end 100 bp reads using Illumina HiSeq 2500. A total of 21,522 genes were analyzed with an average coverage of 85× using Agilent HaloPlex Exome (Agilent Technologies, Santa Clara, CA).

### DGVar gene list

A set of 1604 tumor suppressor genes (TSGs) and oncogenes was curated from the COSMIC database^9^ (version 2018.06.11) and the tumor suppressor gene database^10^ (TSGene 2.0) (https://bioinfo.uth.edu/TSGene/) **(**Supplementary Data 3**)**. For genes with both TSG and oncogene annotations, we treated them as TSGs.

### DGV pipeline (DGVar)

Sequencing reads were processed as previously described^63^, and BAM files were generated. Raw variants were identified using the UnifiedGenotyper variant caller in the Genome Analysis Toolkit v2.5.2^64,65^. The gene harboring each variant and the corresponding effect on transcript products were annotated using SnpEff v4.2^66^ with the pre-built GRCh37.75 database. Reference SNP ID numbers (rs#) were annotated with NCBI dbSNP build 151 ftp://ftp.ncbi.nlm.nih.gov/snp/organisms/human_9606_b151_GRCh37p13/VCF Pathogenicity categories were collected from the NCBI ClinVar database (version 2018.08.05)^66^. Variant frequency in population and two quality filters, the inbreeding coefficient filter and the Variant Quality Score Recalibration (VQSR) filter, were retrieved from the ExAC^66^ database (http://exac.broadinstitute.org) using SnpSift v4.2^67^. We developed **DGVar**, a bioinformatic tool for identifying high confidence putative germline deleterious variants (pDGVs). DGVar applies a series of filtering steps (Supplementary Fig. 1a, b**)**. We filtered variants with low quality (variant quality score lower than 50) or inadequate read coverage (< 10x), SNVs with potential alignment problems (3 or more SNVs in a 10 bp window), variants with a variant allele frequency (VAF) less than 35% that may be attributed to clonal hematopoiesis of indeterminate potential (CHIP) and variants that were commonly observed in healthy populations (> 1% in ExAC). Variant pathogenicity annotations were checked in ClinVar. Variants with likely pathogenic or pathogenic annotations were retained, while variants with likely benign or benign annotations were discarded. ClinVar pathogenic variants associated with non-cancer conditions were manually reviewed and excluded. We then screened TSGs for protein-truncating variants (stop gain or frameshift) that pass the “inbreeding coefficient” and” “VQS” filters in ExAC. To remove platform-related artifacts, variants that were commonly observed (>5%) in the entire WCM cohort were filtered. Variants suspected to be caused by misalignment were removed by manually checking them using IGV. The remaining variants were designated as pDGVs and were used for downstream analysis. After applying these strict filtering criteria, a median of one pDGV per patient was identified (Supplementary Fig. 1a, b). These pDGVs were annotated to indicate if they occur in canonical transcript using snpEff v4.2. A canonical transcript was defined as the longest CDS among the protein-coding transcripts in a gene^66^. The canonical transcripts were annotated using SnpEff v4.2 with its pre-built GRCh37.75 database. A comparison with other pipelines (CharGer and PathoMan)^4,9^ used to detect and annotate germline variants was provided (Supplementary Table 1).

### Functional score prediction using CADD

The deleteriousness of each pDGV was predicted using a Phred-scaled score with Combined Annotation Dependent Depletion (CADD) v1.4^17,18^. To verify that pDGVs in TSGs were more likely to be damaging than protein-truncating germline variants in non-TSGs, a control variant set was prepared from randomly selected 20 protein-truncating germline variants from each patient in non-TSGs. These variants were then scored using CADD as the control set.

### Pathway analysis of pDGVs

To investigate the potential pathways affected by pDGVs, gProfiler^68^ was used to retrieve all pathways that contained pDGV carrying genes. Cancer-associated pathways were selected and scored, based on the likelihood of a pathway being selected by chance, with the following formula:

$$Enrichment{\kern 3pt} score = \log _{10}\left(\!1000 * \frac{{\# genes{\kern 3pt} with{\kern 3pt} DGVs{\kern 3pt} in{\kern 3pt} a{\kern 3pt} pathway}}{{\# genes{\kern 3pt} with{\kern 3pt} DGVs{\kern 3pt} in{\kern 3pt} a{\kern 3pt} patient * \# genes{\kern 3pt} in{\kern 3pt} a{\kern 3pt} pathway}} + 1\!\right).$$

### EthSEQ

The ethnicity of patients in the WCM cohort was inferred using our previously published EthSEQ^13^ method. The reference model built on genotype data from the 1000 Genome Project and the Ashkenazi genome^69^ was chosen. Principal component analysis (PCA) was performed on aggregated genotype data collected from both the reference and WCM individuals. Four conserved ethnic groups: EUR/ASH (Caucasian or Ashkenazi), AFR (African), EAS (East Asian), and SAS (South Asian), were identified by generating the smallest convex sets. Each individual from WCM was assigned to the closest ethnic group. Another refinement step was then performed to differentiate individuals from EUR and ASH groups. We inferred ethnicity for patients in the WCM and TCGA-BLCA cohorts.

### SPARK cohort

We performed ethnicity-matched comparisons to European (10607) and Ashkenazi Jewish (428) individuals in the SPARK cohort. Variants were identified using the DeepVariant caller^70^, and were pre-filtered by removing variants with read coverage less than 8, variant quality score < 30, or VAF < 20%. The variants were initially called on the hg38 genome assembly. For comparison to WCM and TCGA data, we lifted over those variants to hg19 genome assembly using LiftoverVcf in the Picard package (v2.23.0)^71^ and then extracted pDGVs using our variant filtering pipeline DGVar.

### TCGA-BLCA cohort

We downloaded BAM files for germline samples from 398 TCGA bladder cancer (BLCA) patients using the data from the Genomic Data Commons (GDC) legacy data archives using the GDC-client (https://gdc.cancer.gov/about-gdc). We applied our variant filtering pipeline DGVar to the TCGA-BLCA BAM files to retrieve pDGVs using the same steps applied to our WCM-UC cohort. We removed common variants (i.e., found in >5% of the samples) within the TCGA-BLCA cohort since those were likely platform-related artifacts.

### Rare synonymous variants

Rare synonymous variants were defined as synonymous variants having allele frequency <1% in the ExAC database and passing all QC filters used for variant calling. For WCM-UC and TCGA-BLCA cohorts, variant quality score >50, read coverage > = 10, less than 3 SNVs in a 10 bp window, VAF > = 35%, pass the “inbreeding coefficient” and” “VQS” filters in ExAC and occur in <5% individuals in a cohort were used. For the SPARK cohort, variants with read coverage > = 8, variant quality score <30 and VAF > = 20% were used. The same QC filters were applied to both pDGVs and rare synonymous variants.

### pDGV enrichment analysis

To examine whether pDGVs were enriched in the cancer cohorts, we compared the ratio of pDGVs to rare synonymous variants in cancer (WCM and TCGA) and non-cancer (SPARK) cohorts using two-sided Fisher’s exact test. We constructed the contingency table by counting the number of alternative alleles for pDGVs and rare synonymous variants in cancer and non-cancer cohorts. We performed a two-sided Fisher’s exact test using the “fisher.test” function in R. We performed separate ethnicity-matched comparisons using a gene set of 158 genes harboring pDGVs found in the European and Ashkenazi Jewish individuals in the WCM-UC and TCGA-BLCA cohorts. (Supplementary Data 3).

### Comparison with non-urothelial cancer types in the TCGA cohort

We downloaded the filtered variant calls (VCF) released by the TCGA pan-cancer germline study^8^ (https://gdc.cancer.gov/about-data/publications/PanCanAtlas-Germline-AWG). We limited the analysis to individuals with self-reported white ethnicity in the TCGA pan-cancer cohorts and compared to European and Ashkenazi Jewish individuals in the SPARK cohort. We extracted rare synonymous variants and pDGVs based on the filtered variant calls and performed pDGV enrichment analysis by comparing each TCGA cancer cohort with the SPARK non-cancer cohort.

### Comparison with non-urothelial cancer types in WCM cohort

To investigate whether the pDGVs detected in the WCM-UC cohort were present in other WCM cancer cohorts^72^, we selected European and Ashkenazi Jewish patients with prostate cancer (134), kidney cancer (55), glioblastoma (52), colorectal cancer (49), and breast cancer (37). We performed pDGVs enrichment analysis by comparing each WCM cancer cohort with the respective ethnicity-matched SPARK non-cancer cohort.

### Somatic variant detection pipeline

Somatic SNVs and indels were identified using our in-house consensus multi-tool pipeline, which integrated four different somatic variant callers: MuTect2^73^, Strelka^74^, VarScan^75^, and SomaticSniper^76^; these tools identified SNVs in a paired analysis of the tumor and its matched normal sample. Strelka and VarScan were also used to identify indels in the tumor sample. The variants identified from all tools were first aggregated, and only those variants identified by a minimum of two tools were retained for further analysis. The variants were annotated using Oncotator (version 1.9)^77^. The list of variants was further filtered using the following criteria: (a) Variants which did not have a minimum read depth of 10 reads at the corresponding loci were excluded, (b) Variants which did not have a minimum of 3 reads supporting the altered nucleotide were excluded, (c) Variants which did not have a variant allele frequency (VAF) of a minimum 5% in tumor tissue and a maximum of 1% in normal tissue were excluded, (d) Variants that corresponded to the dbSNP^78^ sites were also excluded, unless the specific variants were also reported in the COSMIC database^9^, (e) Technical artifacts, identified in-house for the Haloplex sequencing kit, were also excluded from the final list of mutations. Somatic copy number alterations were identified using the EXaCT-1 somatic pipeline as previously described^63^.

### Analysis of somatic and germline variant co-clusters

Somatic mutation positions obtained from the TCGA PanCancer Atlas studies (32 studies, 10967 samples) were downloaded from cBioportal (https://www.cbioportal.org) and used. Mutation3D^20^ was used to identify co-clusters harboring somatic mutations and pDGVs using the following clustering parameters (i) a minimum cluster size of 3 mutations, (ii) minimum unique amino acid mutations/cluster = 2, (iii) maximum intracluster distance between mutations of 15 Å. The analysis was limited to pDGVs that occurred in genes with available Protein Databank (PDB) structures retrievable by Mutation3D. The analysis used the PDB structure with the highest MPQS score, a composite score calculated by ModBase, and generated from several output measures, including protein coverage, sequence identity, e-value of the alignment, and the discrete optimized protein energy (DOPE) score. The positions of amino acid residues within each cluster in three-dimensional structures were rendered using EzMole 2.1 (http://www.sbg.bio.ic.ac.uk/ezmol/). Lollipop plots were produced using the ProteinPaint tool (https://pecan.stjude.cloud/proteinpaint).

### PCR

Genomic DNA was extracted from the patient’s peripheral blood using the Promega Maxwell LEV Blood DNA Kit (Cat. No AS1290). 100 ng of genomic DNA was amplified using the following primers: F-5′TGGTAAAACACAATCCTTCACG3′, R-5′TTCTTTGGTACCTTTGGATTTGA3′ using standard protocols (Supplementary Table 2). The PCR product was checked on 2% agarose gel to confirm the amplification product. The remaining PCR product was purified using the Qiagen QiAquick PCR cleanup kit (Qiagen USA), and Sanger sequenced (Genewiz USA).

### RT-PCR

RNA was extracted from FFPE macrodissected tumor tissue of WCM049 using the Promega Maxwell LEV RNA FFPE Kit (Cat. No AS1260). 500 ng of RNA extracted from the patient tumor was used to produce the first-strand cDNA using standard protocol using qScript cDNA supermix (Quanta bio. USA). 2ul of cDNA was used in a standard PCR using the following primers F-5′CATCATTCACAATGGGGTGA3” R-5′TCGCCGCAATTCTTTTACTT3” (Supplementary Table 2). 1ul of PCR product was used as a template to re-amplify. PCR product was run on 2% agarose gel to check for amplification. The remaining PCR product was purified using the Qiagen QiAquick PCR cleanup kit (Qiagen USA), and Sanger sequenced (Genewiz. USA).

### Loss of heterozygosity (LOH) analysis

Evaluation of whether LOH events had occurred in genes with pDGVs was performed by calculating the VAFs of pDGVs in tumor samples and comparing it to the VAF observed in the normal sample. In particular, given a patient with pDGVs, joint variant calling was made at the respective pDGV locus in all tumor samples. The VAF was calculated by counting reads supporting reference and alternative alleles in each tumor sample. The VAF was further corrected for tumor purity. This was done by dividing the tumor VAF by the tumor purity and limiting the corrected VAF within the range [0, 1]. Tumor purity was estimated with CLONET^79^, when available, or by pathology review of the H&E slides. CLONET is a computational tool to quantify DNA admixture and ploidy depending on germline heterozygous SNP loci (informative SNPs). This tool can estimate the normal cell admixture and sub-clonal tumor cell population. CLONET was previously used in the TCGA prostate cancer project and was comparable to ABSOLUTE^80^. To investigate whether LOH events were enriched in TSGs, a set of background control variants for each patient was generated by selecting protein-truncating variants in non-TSGs. The background control set was further refined by removing any variants with a VAF < 35% or > 80% in the normal sample since, by definition, LOH occurred in heterozygous loci. Then, joint variant calling was made at those background variants loci in all tumor samples, and VAF was calculated per tumor and corrected for tumor purity. Tumor samples with low tumor purity (<50%) or low coverage of pDGVs (<10 reads) were excluded from the analysis.

### Germline-somatic interactions

The interaction between germline and somatic variants was investigated. First, gene-level events were evaluated by searching for germline and somatic variants that affect the same TSG. Second, this concept was extended to a pathway-level analysis by identifying germline and somatic variants affecting TSG or oncogenes belonging to the same pathway. To screen for pathway-level germline-somatic interaction, pDGVs and somatic variants from each tumor-normal pair were combined, and pathway enrichment analysis was performed using gProfiler^68^. Enriched pathways were determined by selecting those with a p value < 0.05, and pathway-level GSIs were identified by selecting cancer-associated pathways harboring both germline and somatic variants. gProfiler^68^ utilizes three pathway databases: KEGG, Reactome, and WikiPathways. Similar pathways from different source databases were combined. When searching for both gene-level and pathway-level GSIs, variants in TSGs were required to be protein-truncating (loss of function of TSG) and variants in oncogenes to be non-truncating.

### Statistical analysis

The two-sided Fisher’s exact test was used (Fig. 2b, c and Supplementary Fig. 4), odds ratios with 95% intervals were reported. A two-tailed Wilcoxon signed-rank test was used to compare Phred-scaled CADD scores between pDGVs and background variants (Fig. 3a) and compare VAF differences in primary and metastasis tumor samples (Fig. 5c). A two-tailed Kolmogorov–Smirnov test was used to check the tumor-normal VAF difference between pDGVs affecting TSGs and protein-truncating germline variants affecting non-TSGs (Fig. 5b).

### Reporting summary

Further information on research design is available in the Nature Research Reporting Summary linked to this article.

## Supplementary information

Supplementary Information

Description of Additional Supplementary Files

Supplementary Data 1

Supplementary Data 2

Supplementary Data 3

Supplementary Data 4

Supplementary Data 5

Supplementary Data 6

Supplementary Data 7

Supplementary Data 8

Supplementary Data 9

Supplementary Data 10

Supplementary Data 11

Supplementary Data 12

Supplementary Data 13

Supplementary Data 14

Supplementary Data 15

Reporting Summary

## Data Availability

The genomic data supporting the findings of this study are available in the database of Genotypes and Phenotypes (dbGaP). The BAM files and associated sample information are deposited in dbGaP under accession (phs001087.v3.p1). SPARK data are available through https://www.sfari.org/resource/sfari-base/. The COSMIC database is available at https://cancer.sanger.ac.uk/cosmic. The tumor suppressor gene database (TSGene 2.0) is available at https://bioinfo.uth.edu/TSGene/. The dbSNP build 151 is available at ftp://ftp.ncbi.nlm.nih.gov/snp/organisms/human_9606_b151_GRCh37p13/VCF. The NCBI ClinVar database is available at https://www.ncbi.nlm.nih.gov/clinvar/. The ExAC database is available at http://exac.broadinstitute.org. The TCGA pan-cancer germline data is available at https://gdc.cancer.gov/about-data/publications/PanCanAtlas-Germline-AWG.

## Code availability

DGVar is a bioinformatic tool developed for identifying putative deleterious germline variants (pDGVs) from whole-exome sequencing data. Code is available in GitHub at https://github.com/EIPM/dgvar.
