# Supplementary material for: Common germline-somatic variant interactions in advanced urothelial cancer
Source: Nat Commun. 2020 Dec 3;11:6195. doi: 10.1038/s41467-020-19971-8 (PMC7713129; doi:10.1038/s41467-020-19971-8)
Supplement: Supplementary file 1 — Supplementary Information [file 41467_2020_19971_MOESM1_ESM.pdf]

Supplementary Information to:

Common germline-somatic variant interactions in advanced urothelial cancer.

Vosoughi, Zhang, et al.

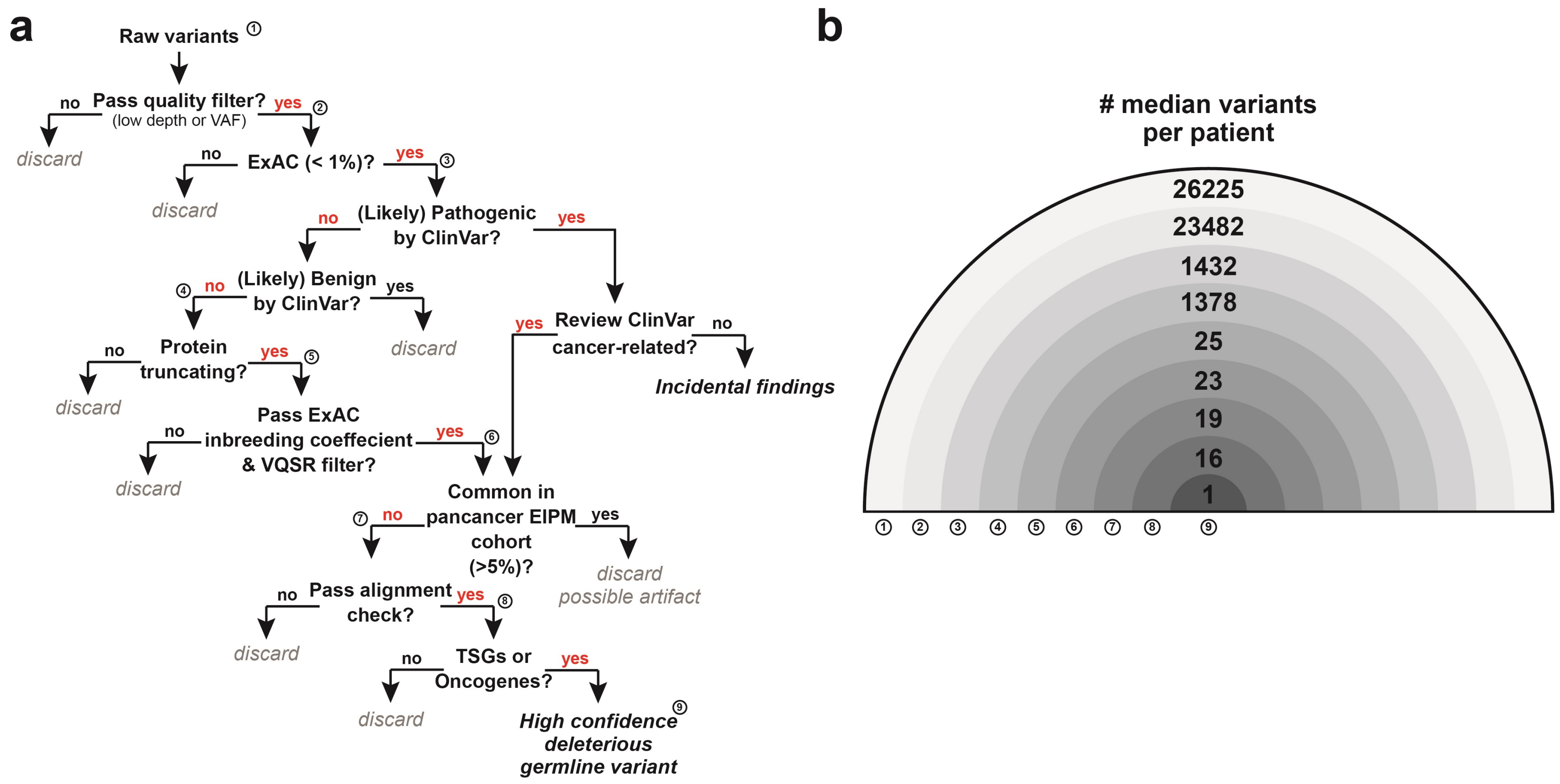

Supplementary Figure 1 DGVar bioinformatic pipeline (a) DGVar steps including quality checks (alignment review and variant quality check), exclusion of common SNPs and platform artifacts, and pathogenicity assessment using ClinVar or predicted protein truncation (b) The number of variants after applying filters to the remaining germline variants after each step.

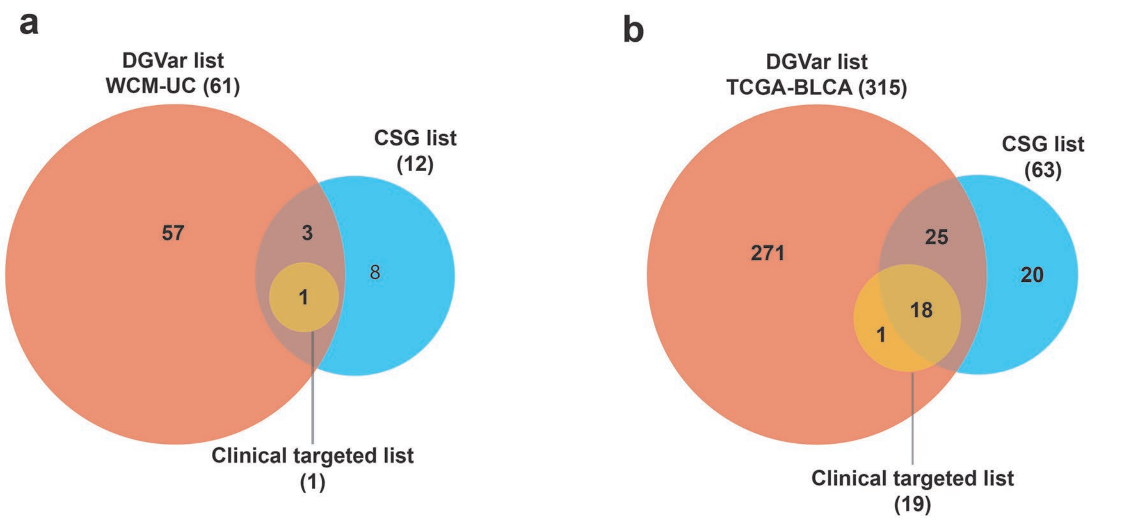

Supplementary Figure 2 Venn Diagram showing the overlapping pDGVs covered by DGVar, a clinical targeted germline sequencing list (https://www.invitae.com/en/physician/tests/01102/#info-panel-resources, Kurian, A. W. et al. *J. Clin. Oncol.* 32, 2001–9, 2014) and a list of CSGs (Huang, K. et al. *Cell* 173, 355-370.e14, 2018) for WCM-UC (n=80) (a) and TCGA-BLCA (n=398) (b). (**Supplementary Data 2 and 4**).

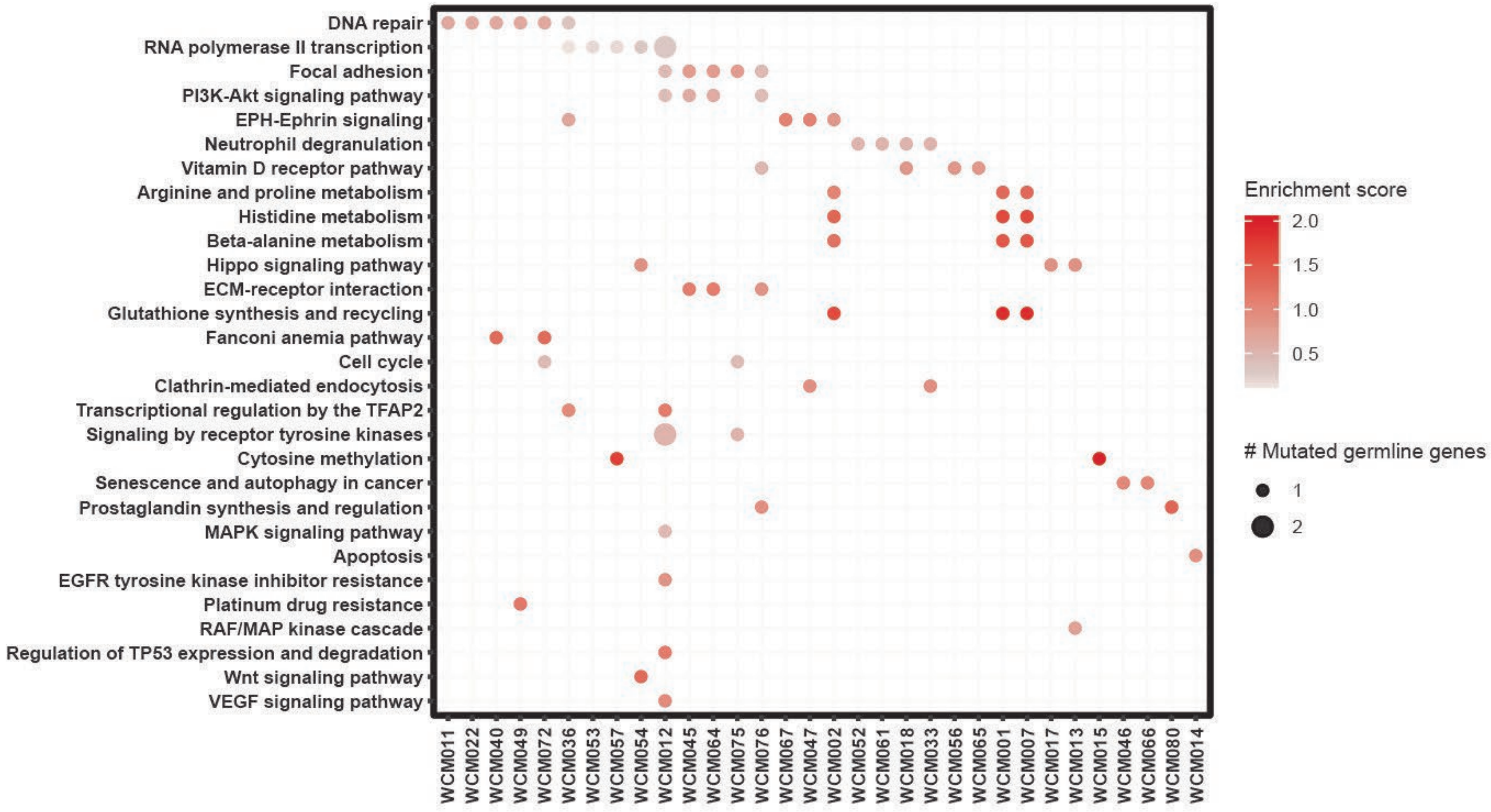

Supplementary Figure 3 DNA repair pathways are enriched in pDGVs. The diameter of each circle represents the number of variants in each affected pathway. Circle color shade represents the enrichment score for each pathway.

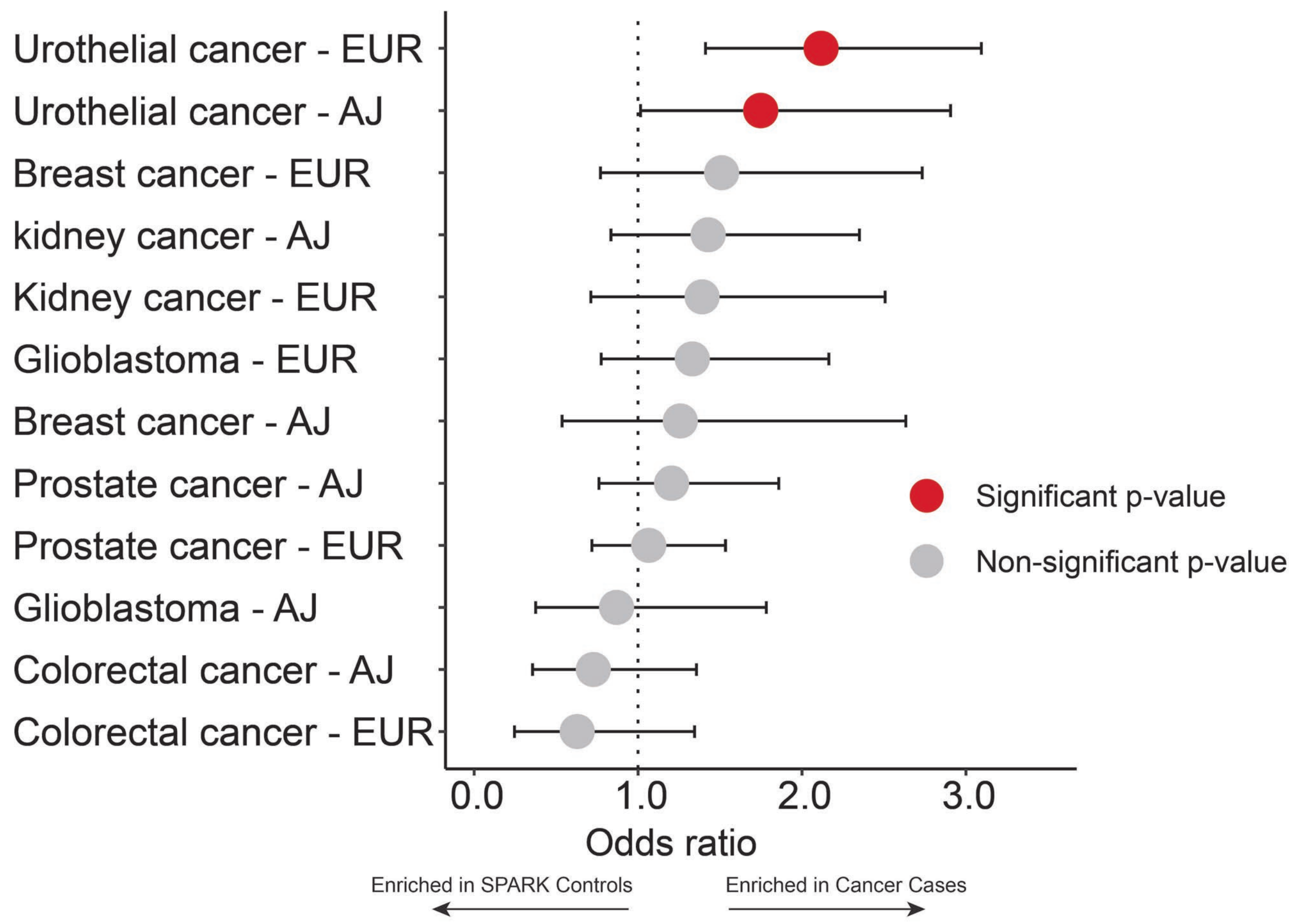

Supplementary Figure 4 The odds ratio of enrichment of pDGVs in a gene set of 158 genes in WCM pan-cancer cohorts (n=399 European or Ashkenazi Jewish individuals) compared with ethnicity-matched non-cancer (SPARK) cohort (n=11,035 European or Ashkenazi Jewish individuals) with two-sided Fisher's exact. Each dot represents the odds ratio (OR), and error bars indicate the 95% confidence intervals (CI). The vertical dotted line represents the odds ratio of 1. Values to the right of this line represents a higher odds ratio of pDGVs to rare synonymous variants in each respective cancer cohort compared to the SPARK non-cancer cohort. Across the WCM cancer cohorts only the urothelial Cancer EUR and AJ subgroups were more likely to harbor pDGVs compared to non-cancer ethnicity matched SPARK cohort. P-value were calculated using two-sided Fisher's exact test. EUR: European, AJ: Ashkenazi Jewish, OR: odds ratio.

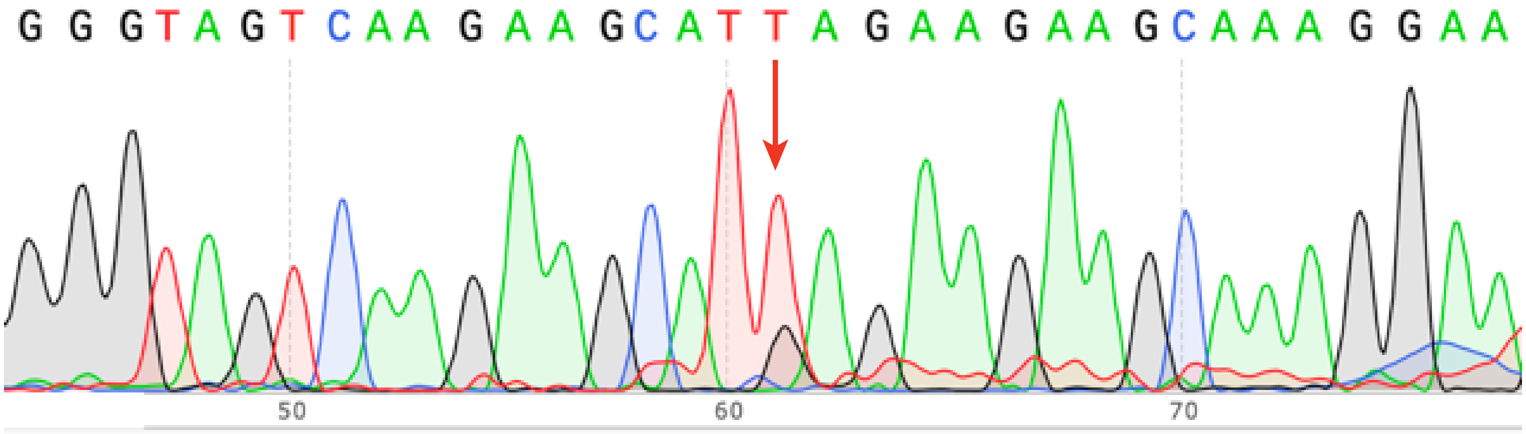

Supplementary Figure 5 RT-PCR and Sanger sequencing from macro-dissected FFPE UC tumor tissue showing the expression of pDGV L200* of *XPA* (c.599T>G) transcript.

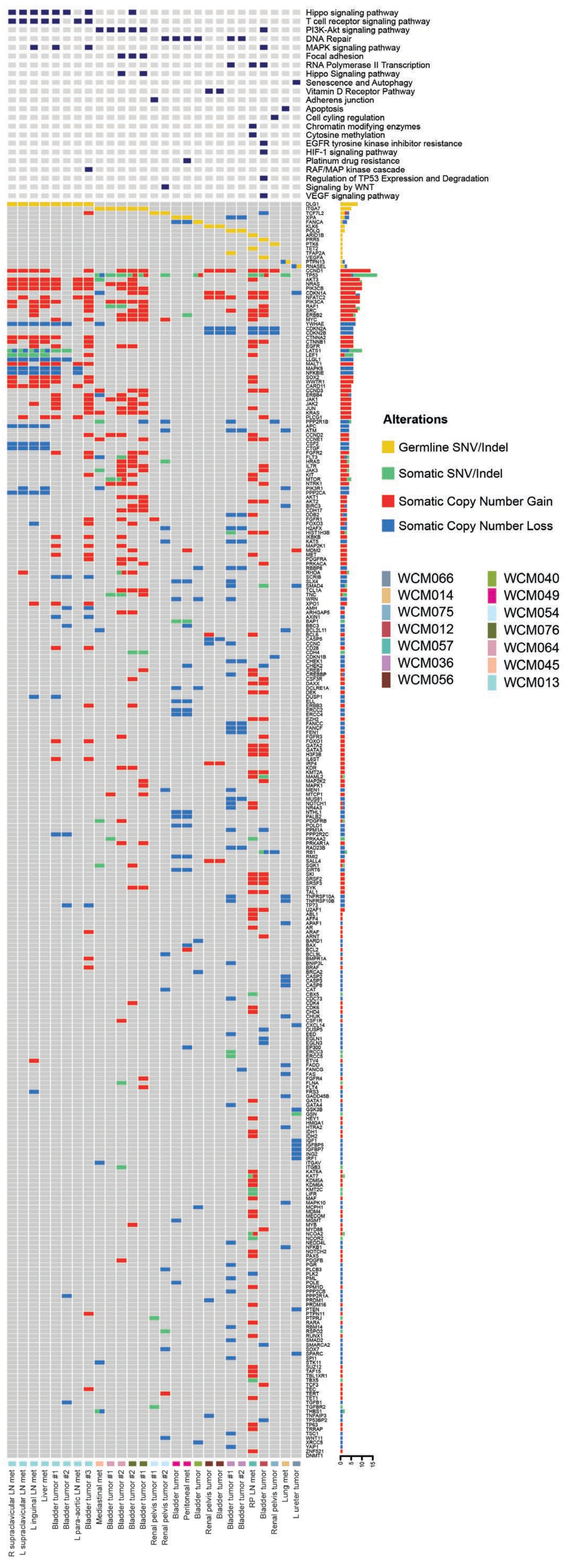

Supplementary Figure 6 Extensive germline-somatic interactions in urothelial carcinoma during tumor progression.

Supplementary Table 1: A comparison between pipelines used to analyze germline variants in patients with urothelial carcinoma

	DGVar (Current Study)	CharGer (Huang et al.)[1]	PathoMan (Carlo et al.)[2]
**Approach**	Discovery of high confidence pDGVs from a large number of background germline variants.<br>Prioritizes variants designated as pathogenic or likely pathogenic by ClinVar or those resulting in truncated proteins encoded by known TSGs.	An automatic variant classification pipeline. Optimizes the clinical interpretation of germline variants within the scope of the ACMG-AMP guidelines in 152 cancer susceptibility genes.	Pathogenic variants (ACMG criteria) by ClinVar. Prioritizes germline Variants in 77 cancer predisposition genes.<br>Sequencing platform: MSK-IMPACT
**Comparison with Non-Cancer Controls**	ExAC and<br>11,035 non-cancer subjects from the SPARK database.	ExAC	ExAC
**Main results**	56% in WCM-UC and 48% in TCGA-BLCA cohort.<br>LOH of pDGVs: 53%	30 pathogenic variants in 7.3% in the TCGA-BLCA cohort (17 of those variants annotated as P/LP by ClinVar and 8% in pancancer TCGA	86 P/LP variants in 80/586 (14%) patients<br>LOH of DDR genes: 33.3%

pDGVs: putative deleterious germline variants, TSGs: tumor suppressor genes, ACMG: American College of Medical Genetics, LOH: loss of heterozygosity, DRR: DNA damage repair genes. P/PL: pathogenic/likely pathogenic.

Supplementary Table 2: Primers used in this study

Genes	Sense/Primer-F（5'-3'）	Antisense/Primer-R（5'-3'）	Usage
XPA	TGGTAAAACACAATCCTTCACG	TTCTTTGGTACCTTTGGATTTGA	PCR
XPA	CATCATTCACAATGGGGTGA	TCGCCGCAATTCTTTTACTT	RT-PCR

Supplementary References

1. Huang, K. et al. Pathogenic Germline Variants in 10,389 Adult Cancers. Cell 173, 355-370.e14 (2018).
2. Carlo, M. I. et al. Cancer Susceptibility Mutations in Patients With Urothelial Malignancies. J. Clin. Oncol. 38, 406–414 (2020).
