# Supplementary material for: Common germline-somatic variant interactions in advanced urothelial cancer
Source: Nat Commun. 2020 Dec 3;11:6195. doi: 10.1038/s41467-020-19971-8 (PMC7713129; doi:10.1038/s41467-020-19971-8)
Supplement: Supplementary file 2 — Description of Additional Supplementary Files [file 41467_2020_19971_MOESM2_ESM.pdf]

Description of Additional Supplementary Files

Title: Supplementary Data 1

Description: Clinical characteristics of patients form WCM-UC, patients and matched tumor-normal samples used in this analysis

Title: Supplementary Data 2:

Description: List of the 61 pDGVs identified in the WCM-UC cohort.

Title: Supplementary Data 3

Description: List of the 1604 cancer-related genes used in DGVar, list of the 152 Cancer Susceptibility genes (CSGs) curated by Huang et al. and list of the 47 genes in the Invitae targeted panel.

Title: Supplementary Data 4

Description: List of the 315 pDGVs identified in the TCGA-BLCA cohort.

Title: Supplementary Data 5:

Description: List of pDGVs that were recurrent (occurred in two or more patients) within WCM-UC cohort, list of recurrent pDGVs (occurring in two or more patients) within TCGA-BLCA cohort, recurrent pDGVs occurring in two or more patients in either WCMUC and/or TCGA-BLCA cohort, shared pDGVs occurring in at least one patient in both WCM-UC and TCGA-BLCA cohorts.

Title: Supplementary Data 6:

Description: Genes harboring pDGVs in WCM-UC cohort and reported in COSMIC Cancer Gene Census list, genes harboring pDGVs in TCGA-BLCA cohort and reported in COSMIC Cancer Gene Census list, list of genes harbouring pDGVs in WCM-UC cohort and reported to harbor germline variants in other cancers in COSMIC Cancer Gene Census, list of 24 genes harbouring pDGVs in TCGA-BLCA cohort and reported to have germline variants in the COSMIC Cancer Gene Census.

Title: Supplementary Data 7

Description: Enrichment of pDGVs in the WCM-UC cohort compared to the SPARK cohort.

Title: Supplementary Data 8:

Description: Enrichment of pDGVs in the TCGA-BLCA cohort compared to the SPARK cohort.

Title: Supplementary Data 9

Description: Enrichment of pDGVs in other TCGA cancer cohorts.

Title: Supplementary Data 10

Description: Enrichment of pDGVs in other WCM cancer cohorts.

Title: Supplementary Data 11

Description: The average the Phred scaled combined annotation dependent depletion (CADD) scores of the identified pDGVs in WCM-UC.

Title: Supplementary Data 12

Description: Co-clustering analysis of pDGVs identified in WCM-UC cohort with TCGA pancancer somatic mutations for the KLK6, EPHB6, XPA, TRIM32, PINX1 clusters.

Title: Supplementary Data 13

Description: Loss of heterozygosity analysis of tumor samples with sufficient purity (>50%).

Title: Supplementary Data 14

Description: Corrected VAFs of pDGVs in the primary and metastatic tumor sample pairs.

Title: Supplementary Data 15

Description: Enriched pathways in tumor samples involving both pDGVs and somatic alterations.
