# Supplementary material for: Common germline-somatic variant interactions in advanced urothelial cancer
Source: Nat Commun. 2020 Dec 3;11:6195. doi: 10.1038/s41467-020-19971-8 (PMC7713129; doi:10.1038/s41467-020-19971-8)
Supplement: Supplementary file 18 — Reporting Summary [file 41467_2020_19971_MOESM18_ESM.pdf]

nature research

Corresponding author(s): Bishoy Faltas

Last updated by author(s): Nov 4, 2020

Reporting Summary

Nature Research wishes to improve the reproducibility of the work that we publish. This form provides structure for consistency and transparency in reporting. For further information on Nature Research policies, see Authors & Referees and the Editorial Policy Checklist .

Statistics

For all statistical analyses, confirm that the following items are present in the figure legend, table legend, main text, or Methods section.

n/a	Confirmed	
☐	☒	The exact sample size (*n*) for each experimental group/condition, given as a discrete number and unit of measurement
☐	☒	A statement on whether measurements were taken from distinct samples or whether the same sample was measured repeatedly
☐	☒	The statistical test(s) used AND whether they are one- or two-sided<br>*Only common tests should be described solely by name; describe more complex techniques in the Methods section.*
☐	☒	A description of all covariates tested
☐	☒	A description of any assumptions or corrections, such as tests of normality and adjustment for multiple comparisons
☐	☒	A full description of the statistical parameters including central tendency (e.g. means) or other basic estimates (e.g. regression coefficient) AND variation (e.g. standard deviation) or associated estimates of uncertainty (e.g. confidence intervals)
☐	☒	For null hypothesis testing, the test statistic (e.g. *F*, *t*, *r*) with confidence intervals, effect sizes, degrees of freedom and *P* value noted<br>*Give P values as exact values whenever suitable.*
☒	☐	For Bayesian analysis, information on the choice of priors and Markov chain Monte Carlo settings
☒	☐	For hierarchical and complex designs, identification of the appropriate level for tests and full reporting of outcomes
☒	☐	Estimates of effect sizes (e.g. Cohen's *d*, Pearson's *r*), indicating how they were calculated

Our web collection on statistics for biologists contains articles on many of the points above.

Software and code

Policy information about availability of computer code

Data collection	Code used to interrogate the germline variants in this study is available at https://github.com/EIPM/dgvar
Data analysis	Code used to analyze the data in this study is available at https://github.com/EIPM/dgvar. The following software/tools/algorithms/packages were used:<br>Genome Analysis Toolkit v2.5.2, SnpEff v4.2, SnpSift v4.2, Combined Annotation Dependent Depletion (CADD) v1.4, Picard package v2.23.0, EthSEQ, CLONET, gProfiler, Mutation3D, EzMole 2.1, ProteinPaint, MuTect2, Strelka, VarScan, SomaticSniper, and Oncotator (version 1.9)

For manuscripts utilizing custom algorithms or software that are central to the research but not yet described in published literature, software must be made available to editors/reviewers. We strongly encourage code deposition in a community repository (e.g. GitHub). See the Nature Research guidelines for submitting code & software for further information.

Data

Policy information about availability of data

All manuscripts must include a data availability statement. This statement should provide the following information, where applicable:

- Accession codes, unique identifiers, or web links for publicly available datasets
- A list of figures that have associated raw data
- A description of any restrictions on data availability

The genomic data supporting the findings of this study are available in the database of Genotypes and Phenotypes (dbGaP). The BAM files and associated sample information are deposited in dbGaP under accession phs001087.v3.p1). SPARK data are available through https://www.sfari.org/resource/sfari-base/. The COSMIC database is available at https://cancer.sanger.ac.uk/cosmic. The tumor suppressor gene database (TSGene 2.0) is available at https://bioinfo.uth.edu/TSGene/. The dbSNP build 151 is available at ftp://ftp.ncbi.nlm.nih.gov/snp/organisms/human_9606_b151_GRCh37p13/VCF. The NCBI ClinVar database is available at https://www.ncbi.nlm.nih.gov/clinvar/. The ExAC database is available at http://exac.broadinstitute.org. The TCGA pan-cancer germline data is available at https://gdc.cancer.gov/about-data/publications/PanCanAtlas-Germline-AWG.

Field-specific reporting

Please select the one below that is the best fit for your research. If you are not sure, read the appropriate sections before making your selection.

- [x] Life sciences
- [ ] Behavioural & social sciences
- [ ] Ecological, evolutionary & environmental sciences

For a reference copy of the document with all sections, see nature.com/documents/nr-reporting-summary-flat.pdf

Life sciences study design

All studies must disclose on these points even when the disclosure is negative.

Sample size	The study included 80 UC patients from Weill Cornell Medicine (WCM) and 398 patients from The Cancer Genome Atlas (TCGA). WCM were used as the discovery cohort and the TCGA UC cohort was used as a validation cohort. Cohort size was determined based on the availability of whole-exome sequencing data.
Data exclusions	no data were excluded from analysis
Replication	We validated our findings using DGVar to analyze germline WES data from 398 TCGA UC cohort and compared to 11,035 non-cancer subjects from SPARK dataset.
Randomization	No randomization was performed. This was a cross-sectional study of putative deleterious germline variants in patients with advanced urothelial cancer. There was no assignment to interventional groups requiring randomization.
Blinding	Blinding was not applicable to our study which was a cross-sectional study of putative deleterious germline variants in patients with advanced urothelial cancer and did not apply experimental interventions to the studied subjects requiring blinding.

Reporting for specific materials, systems and methods

We require information from authors about some types of materials, experimental systems and methods used in many studies. Here, indicate whether each material, system or method listed is relevant to your study. If you are not sure if a list item applies to your research, read the appropriate section before selecting a response.

Materials & experimental systems

n/a	Involved in the study	
☒	☐	Antibodies
☒	☐	Eukaryotic cell lines
☒	☐	Palaeontology
☒	☐	Animals and other organisms
☐	☒	Human research participants
☒	☐	Clinical data

Methods

n/a	Involved in the study	
☒	☐	ChIP-seq
☒	☐	Flow cytometry
☒	☐	MRI-based neuroimaging

Human research participants

Policy information about studies involving human research participants

Population characteristics	We included 80 patients with histologically-proven urothelial carcinoma with median age of 70 years (range 43-85). Most patients were male 59/80 (74%) and sixty-six patients (82.5%) had metastatic disease. The majority 61/80 (76%) had a history of smoking, 39 patients (49%) had a history of a second non-UC primary cancer, and 40 patients (50%) had a family history of cancer in at least one first-degree relative. Computational genomic ethnicity analysis using EthSEQ2 showed a high representation of European 72/80 (90%) ancestry in our cohort, of which 27/80 (34%) were Ashkenazi Jewish
Recruitment	Peripheral blood, buccal swab samples, and in one patient, normal liver tissue were collected for germline DNA extraction from 80 patients diagnosed with high-grade urothelial carcinoma (HGUC). Fresh frozen and formalin-fixed paraffin-embedded (FFPE) tissue from biopsies, cystectomy, and nephroureterectomy specimens from HGUC patients were collected. Our urothelial cancer cohorts had a high representation of patients with European ancestry, so we used ethnicity-matched SPARK-EUR and SPARK-AJ non-cancer cohorts for comparison.
Ethics oversight	All experimental procedures were carried out in accordance with approved guidelines and were approved by the Institutional Review Boards at Weill Cornell Medicine. Patients recruited to this study signed informed consent under IRB-approved protocols: Weill Cornell Medicine (WCM)/New York-Presbyterian (NYP) IRB protocols for Tumor Biobanking—0201005295, GU tumor Biobanking—1008011210, Urothelial Cancer Sequencing—1011011386, Comprehensive Cancer Characterization by Genomic and Transcriptomic Profiling—1007011157 and Precision Medicine—1305013903).

Note that full information on the approval of the study protocol must also be provided in the manuscript.
